# Supplementary material for: Probing the edge-related properties of atomically thin MoS2 at nanoscale
Source: Nat Commun. 2019 Dec 5;10:5544. doi: 10.1038/s41467-019-13486-7 (PMC6895227; doi:10.1038/s41467-019-13486-7)
Supplement: Supplementary file 1 — Supplementary information [file 41467_2019_13486_MOESM1_ESM.pdf]

Supplementary Information

Probing the edge-related properties of atomically thin MoS_2 at nanoscale

Teng-Xiang Huang et al.

Supplementary Note 1. Doping effect from Au substrate and Au AFM-TERS tip

Au substrates and Au tips were employed in this work. However, the doping effect of the substrate can be neglected. From the AFM height curve (Fig. 3a) and AFM images (Supplementary Fig. 1a-d), we can find that a thin ice-like water layer (0.3~0.6 nm) exists between MoS_2 and the Au film, which can block the doping effect from the Au substrate. Furthermore, as shown in Supplementary Fig. 1e, the A_{1g} peak of monolayer MoS_2 on the Au substrate shows almost the same frequency as that on silica, indicating that no doping occurred between MoS_2 and Au substrate. Only after the removal of the water layer, the Au substrate could directly interact with MoS_2, leading to the decrease of the PL intensity (Supplementary Fig. 1f) and the upshift of the A_{1g} peak (Supplementary Fig. 1g).

In the case of the tip, although there is still a small gap (~0.5 nm) existing between the tip and MoS_2 in the contact mode, we did find the charge transfer between tips and MoS_2 due to different work functions of tip materials (Au or Ag) and MoS_2 (Φ_{Ag} (4.3 eV) < Φ_{MoS2} (4.7 eV) < Φ_{Au} (5.1 eV))[1] and the strong surface plasmon resonance at the tip apex. As shown in Supplementary Fig. 2, when an Au tip approached MoS_2, the electrons were transferred from MoS_2 to the Au tip, leading to the p-doping of MoS_2 and the upshift of the A_{1g} peak. Conversely, when an Ag tip approached MoS_2, the electrons were transferred from the Ag tip to MoS_2, leading to the n-doping of MoS_2 and the downshift of the A_{1g} peak. When the tips were coated with a thin silica layer, no doping effect was observed. Although charge transfer between the tip and MoS_2 existed in the TERS measurement, it will not influence the final conclusion since the tip did have a similar doping effect on the edge and the basal plane.

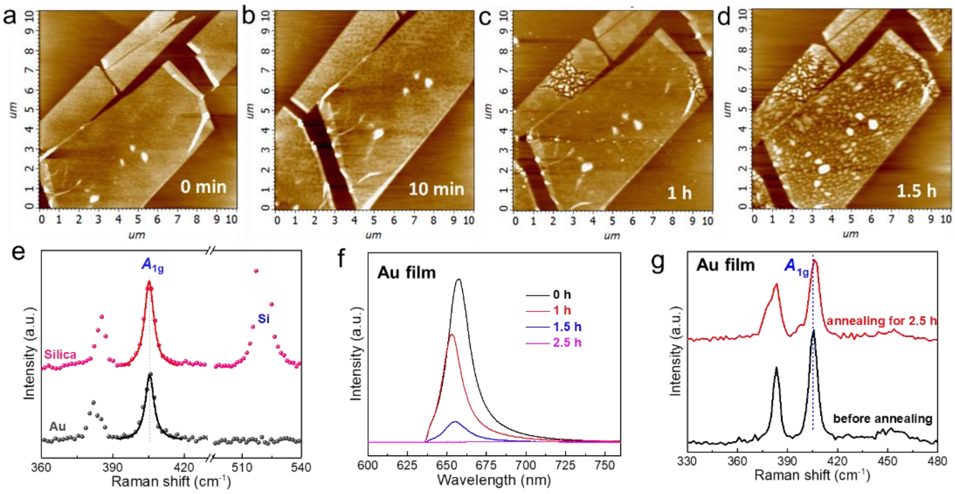

Supplementary Figure 1 The presence of the water layer between the monolayer MoS_2 and Au film prevents the doping effect of Au substrate on MoS_2. (**a-d**) AFM images of monolayer MoS_2 on the Au film before and after annealing at 80 ^oC for a different time. (**e**) Raman spectra of monolayer MoS_2 on the Au film and silica substrate before annealing. The excitation laser wavelength was 532 nm and the laser power was 0.1 mW, and the acquisition time was 10 s. Photoluminescence (**f**) and Raman (**g**) spectra of monolayer MoS_2 on the Au film before and after annealing at 80 ^oC for a different time. The excitation laser wavelength in PL measurements was 632.8 nm with a laser power of 0.03 mW and the acquisition time was 1 s. The excitation laser wavelength in Raman measurements was 532 nm with a laser power of 0.3 mW and the acquisition time was 30 s.

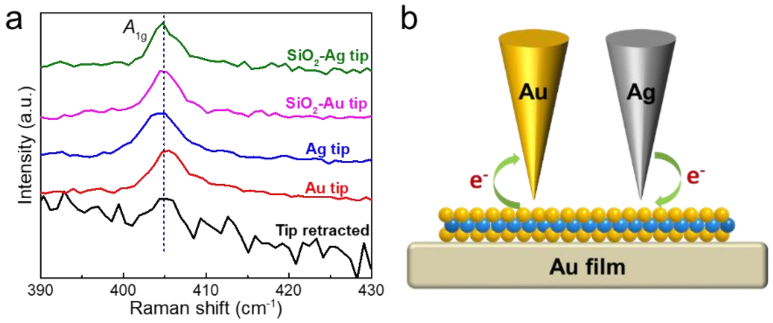

Supplementary Figure 2 Demonstration of the charge transfer between TERS tips and MoS_2. (**a**) Peak positions of the A_{1g} mode after the approaching of different tips. (**b**) Schematic of the charge transfer between the tip and MoS_2. The excitation laser was 632.8 nm.

Supplementary Note 2 Deconvolution of EM field

2.1 Fitting of the TERS intensity profile

The 396 cm^{-1} intensity profile (Fig. 3c, red dots) can be deconvoluted into two parts and expressed as:

$$I = \exp\left(-\frac{(x-a)^2}{b^2}\right) \otimes \left(g(x) + e\,\delta(x)\right) \qquad (1)$$

$$g(x) = \begin{cases} c & x < 0 \\ d & x \geq 0 \end{cases} \qquad (2)$$

where the Gaussian expression, $\exp\left(-\frac{(x-a)^2}{b^2}\right)$, in Equation 1 is the transversal intensity distribution of the electromagnetic (EM) field under the tip apex and the $g(x)$ is a step function. During the TERS imaging, the Gaussian distributed EM field of the tip is progressively scanned over the step-like MoS_2 and the TERS intensity can be considered as the convolution of EM field and a step function, $g(x)$ (Supplementary Fig. 3, blue line). Both c and d in $g(x)$ are constant with different values. Since a small region (~1.8 nm) of pristine MoS_2 near the edge undergoes band bending and gives enhanced TERS signal, such a double resonance Raman scattering (DRRS) band bending related signal can be expressed as the convolution of the EM field with a $e\delta(x)$ function (Supplementary Fig. 3, red line). The $\delta(x)$ can be defined as constants because the band bending region (~1.8 nm) is much smaller than the normal TERS spatial resolution in air (7~10 nm). Therefore, $\delta(x) = 1$, when $0 < x \leq 1.8$, and $\delta(x) = 0$, when $x \leq 0$ or $x > 1.8$. After the data fitting, we obtain the five coefficients (with 95% confidence bounds) as a = -0.3852±0.2760, b = 4.572±0.383, c = 0.000675±0.00378, d = 0.01287±0.00542, e = 2.279±0.153. The final fitted result of the 396 cm^{-1} the intensity profile is shown in Fig. 3 as the red solid line. The similar fitting process was applied to the 406 cm^{-1} intensity profile and the fitted result is shown in Fig. 3 as the blue solid line.

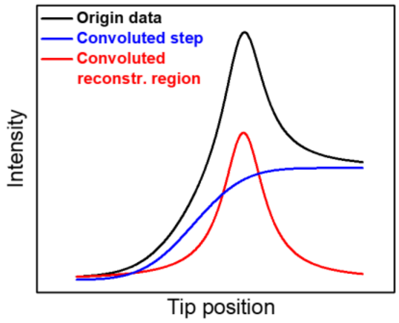

Supplementary Figure 3 The schematic diagram of the fitting principle. See the above

paragraph for the detailed meaning of each curve.

2.2. Calculation of the size of the electronic transition region

Similar to Equation 1, the intensity profile of the 406 cm^{-1} peak (Fig. 3c, blue dots) can be expressed as:

$$I = \exp\left(-\frac{(x-a)^2}{b^2}\right) \otimes (g'(x) + f(x)) \qquad (3)$$

$$g'(x) = \begin{cases} c' & x < 0 \\ d' & x \geq 0 \end{cases} \qquad (4)$$

where a = -0.3852±0.2760 and b = 4.572±0.383 as mentioned above. $g'(x)$ is a step function and the $f(x)$ is the original enhanced TERS intensity profile of the electronic transition region. As shown in the Supplementary Fig. 4a, the black line is the experimental intensity profile of 406 cm^{-1} peak and the green Gaussian curve is the transversal intensity distribution of the EM field calculated in Supplementary Note 1. The red line is the convolution of EM field and $g'(x)$, where c' = 0.002226±0.008223 and d' = 0.05891±0.00272 after calculation. The blue line is the subtraction of the convoluted step function (red line) from the experimental intensity profile (black line). Therefore, it is the convolution of the Gaussian-distributed EM field with the original enhanced TERS intensity of the electronic transition region ($f(x)$). After the deconvolution of the intensity distribution of the EM field, the original enhanced TERS intensity of the electronic transition region ($f(x)$) is shown in Supplementary Fig. 4b. Therefore, the size of the electronic transition region is estimated to be ~10 nm by a 10% contrast analysis[2, 3]. It is worth to note that different from the maximum intensity of LA(**M**)+TA(**M**) mode (the largest lattice reconstruction) located at the edge site, the maximum intensity of A_{1g} mode (the lowest electron density) appears inside the pristine MoS_2 and is located in 5 nm away from the edge.

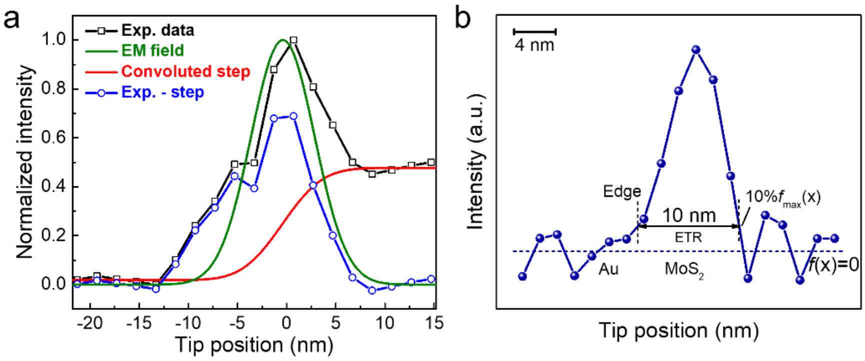

Supplementary Figure 4 Calculation of the size of the electronic transition region (ETR). (a) Calculation of the electronic transition region by using the TERS intensity profile of 406 cm^{-1} peak, as stated in the above paragraph. The green Gaussian curve is the transversal intensity distribution of the EM field. The black and the red lines are the experimental intensity profile and the convoluted step function curve, respectively. The blue line, which is the subtraction of the red line from the black line, is the convolution of the Gaussian-distributed EM field with the original enhanced TERS intensity of the electronic transition region ($f(x)$). (**b**) The enhanced TERS intensity profile of the electronic transition region, which is calculated by the deconvolution of the intensity distribution of the EM field from the blue line shown in Supplementary Fig. 4a.

2.3. FWHM analysis of TERS intensity profile of 396 cm^{-1} peak

In order to estimate the length of the band bending that DRRS involved, it is necessary to have both the TERS intensity profile of 396 cm^{-1} peak and the spatial distribution of the EM field. As shown in Supplementary Fig. 5, the red line is the experimental fitted intensity profile of 396 cm^{-1} peak and the green curve is the convolution result of the EM field and the step function. Subtracting the red line with the green line, we obtain the blue line, which is the convolution result of the Gaussian-distributed EM field with the original enhanced TERS intensity of the band bending region. Since the full width at half maximum (FWHM = 7 nm) of the blue line is equal to the typical AFM-based TERS spatial resolution (~ 7 nm[4], which is estimated by the FWHM analysis of the transversal intensity distribution of EM field) obtained in our lab, we can rationalize that the length of the band bending region should be much smaller than that of the TERS spatial resolution (7 nm). Indeed, theoretical calculation reveals that the band bending region, which DRRS involved, of the pristine MoS_2 induced by the edge, is only about two unit cells of 1.8 nm (shown in Supplementary Fig. 11).

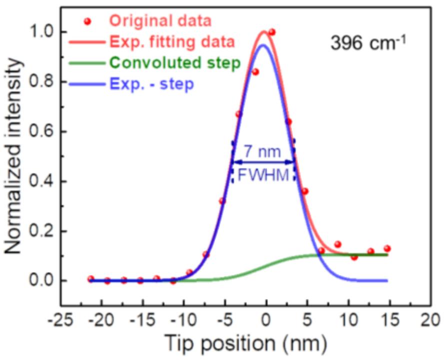

Supplementary Figure 5 FWHM analysis of TERS intensity profile of 396 cm^{-1} peak. The red line is the fitted profile of experimental TERS data (red dot). The green line is the convolution result of the EM field and the step function. The blue line is the convolution result of the Gaussian-distributed EM field with the original enhanced TERS intensity of the band bending region.

Supplementary Note 3 Calculation of the electronic properties of defects

3.1 Calculation models of defects in MoS_2

The theoretical simulation methods have been described in the method part of the main text. We employed nanoribbons with armchair (ANR) and zigzag (ZNR) edges and different widths (1~3 nm, n=8~20) (Supplementary Fig. 6a-c) to estimate the edge effect on electronic and vibrational properties. In order to investigate the wrinkle and step in MoS_2, the 1L wrinkle in MoS_2 is simplified into 1L MoS_2 with 0.4% uniaxial strain (Supplementary Fig. 6d), according to the frequency shift of strain-sensitive $E_{2g}{}^1$ mode (Supplementary Fig. 7). The 1L-2L step can be constructed by a vertical combination of 1L edge and 1L basal plane, where 1L edge can be simplified as a nanoribbon. Consequently, the 1L-2L step is modeled by a combination of 1L ANR and 1L basal plane. To avoid the interaction between periodic ANR, lattice spacing along $\boldsymbol{y}$ direction is larger than 10 Å, as shown in Supplementary Fig. 6e.

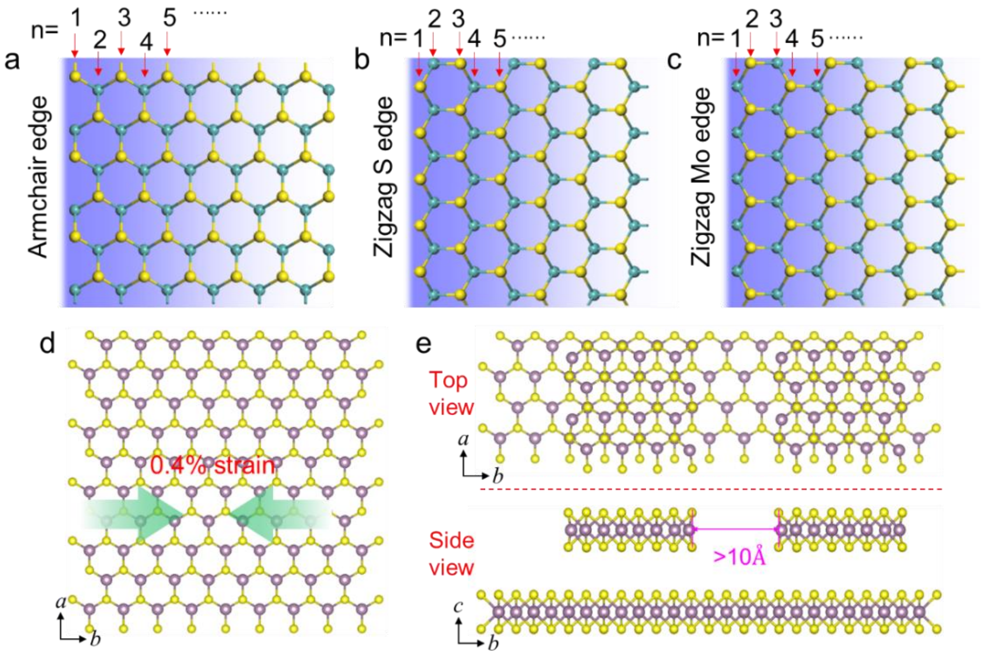

Supplementary Figure 6 Schematic models of nanoribbons with the armchair edge (**a**), zigzag S edge (**b**), zigzag Mo edge (**c**), and the wrinkle (**d**) and 1L-2L step (**e**) in MoS_2.

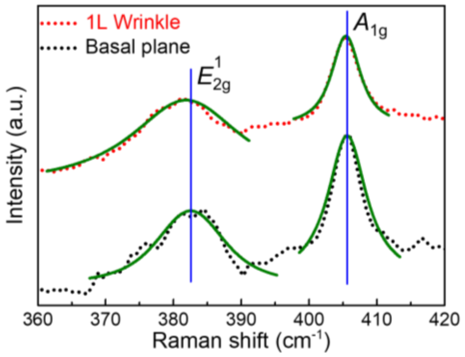

Supplementary Figure 7 Lorentz fitting of E^1_{2g} and A_{1g} modes of the TERS spectra when the tip approached the basal plane and wrinkle in a monolayer MoS_2. The peak position of A_{1g} mode remains constant, while the peak position of E^1_{2g} mode shifts downward for about 1 cm^{-1} at the wrinkle and this mode does not split into two singlet sub-bands, indicating a 0.4% tensile strain in the wrinkle[5, 6].

3.2 Calculation of electronic band structures of the armchair nanoribbon

To reveal the edge effect on the energy of c_K and c_Q states, nanoribbon of MoS_2 with the armchair edge (ANR) is employed, where the width of ANR is defined in Supplementary Fig. 6a. We calculated the band structure of pristine MoS_2 and 1L ANR (with a width of ~6.2 nm, n=39, 1L 39ANR) with the fat-band analysis as well as the charge density of related states, as shown in

Supplementary Fig. 8. It clearly shows that some additional bands exist in the gap of 1L ANR, which might be attributed to edge states due to dangling bonds with one-dimensional translation symmetry[7]. To further investigate $c_{\mathbf{K}}$ and $c_{\mathbf{Q}}$ states, charge density isosurfaces of specified states are presented in Supplementary Fig. 8d and e. The charge densities of conduction band minimum (CBM, $c_{\mathbf{K}1}$) and valence band maximum (VBM, $v_{\mathbf{K}}$) are localized near the edge, indicating that CBM, VBM, and the band gap of ANR are determined by edge states. Furthermore, the charge densities of $c_{\mathbf{K}(1\text{-}6)}$ and $v_{\mathbf{K}(1\text{-}6)}$ states also indicate that bands in the gap of bulk bands originate from the edge. Bands above edge bands result from the folded and degenerated bands of "bulk" MoS_2 (here, "bulk" MoS_2 corresponds to pristine 1L MoS_2).

As discussed in the main text, the DRRS process of LA(**M**)+TA(**M**) mode involves $c_{\mathbf{K}}$ and $c_{\mathbf{Q}}$ states, and the possibility of the related Raman process can be largely enhanced for the large energy difference between $c_{\mathbf{K}}$ and $c_{\mathbf{Q}}$ states ($E_{\mathbf{CK}}$-$E_{\mathbf{CQ}}$). For ANR, the Brillouin zone (BZ) can be obtained from folded BZ of the unit cell, as shown in Supplementary Fig. 8a. **K** and **Q** points in BZ of the unit cell are folded into the center (**Γ**) and the edge (**X**) of BZ of ANR, respectively. Electronic bands are consequently folded. It is noted that each electronic state in the translationally periodic crystal can be presented by bulk Bloch functions. In ANR, the translational periodicity is partially removed, and the electronic state can be described by the superposition of the bulk Bloch functions of the underlying perfect crystals. These would lead to the band folding, and the band folding induced quasi-direct transitions are trivial[8]. Therefore, the DRRS process in ANR mainly involves folded $c_{\mathbf{K}}$ and $c_{\mathbf{Q}}$ states, which provides the possibility to investigate LA(**M**)+TA(**M**) mode at edge.

To identify folded $c_{\mathbf{K}}$ and $c_{\mathbf{Q}}$ states in ANR, the fat-band analysis and charge density isosurfaces of pristine MoS_2 and ANR are employed, as presented in Supplementary Fig. 8b-e. According to the fat-band analysis, the $c_{\mathbf{K}}$ and $c_{\mathbf{Q}}$ states are mainly composed of d_{z^2} and $d_{x^2-y^2}$ orbitals of Mo atoms, respectively. The charge density isosurfaces of $c_{\mathbf{K}}$ and $c_{\mathbf{Q}}$ states in pristine MoS_2 are similar to that of 1L 39ANR at **K** and **Q** points, respectively, which is in agreement with the above analysis about BZ folding. To exhibit direct comparison, the charge density of $c_{\mathbf{K}}$ and $c_{\mathbf{Q}}$ states in supercell repeated along zigzag (***b***) direction is also calculated, as presented in Supplementary Fig. 8d. It is obvious that the charge densities of $c_{\mathbf{K}7\text{-}10}$ states are similar to that of $c_{\mathbf{K}}$ state, indicating that $c_{\mathbf{K}7\text{-}10}$ states in 1L 39ANR results from folded and degenerated $c_{\mathbf{K}}$ states, and the $c_{\mathbf{Q}}$ state can also be

identified. Therefore, $c_{\mathbf{K}}$ and $c_{\mathbf{Q}}$ states in ANR can be identified by fat-band analysis and charge density, and LA(**M**)+TA(**M**) mode at edge can be estimated by edge effect on $c_{\mathbf{K}}$ and $c_{\mathbf{Q}}$ states.

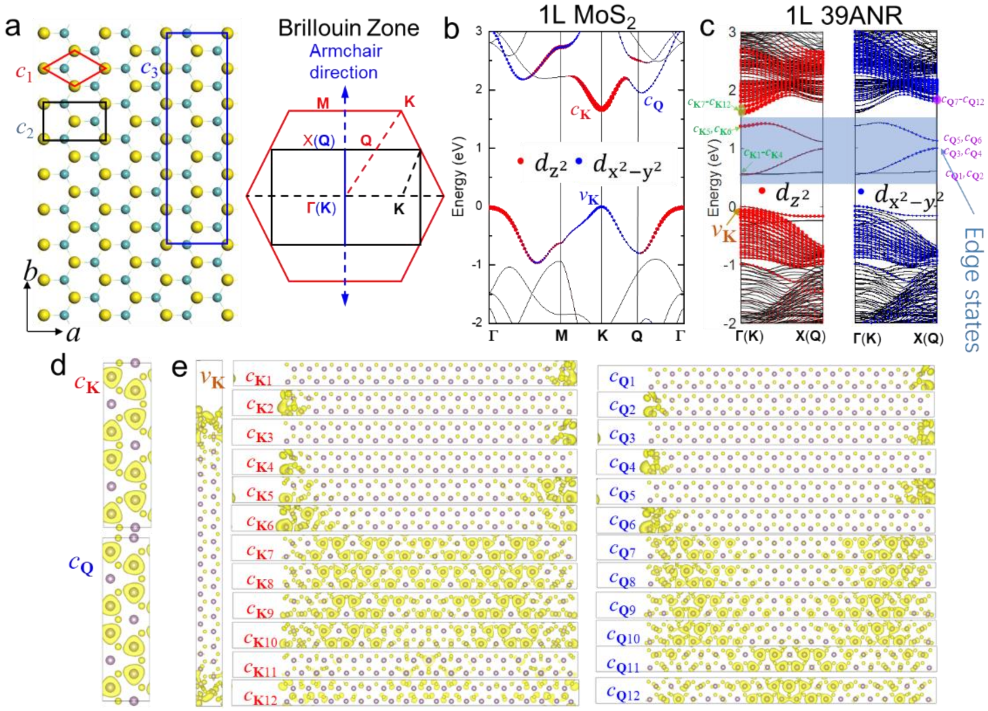

Supplementary Figure 8 DFT calculation the electronic band structure of MoS_2 and 1L39ANR. (**a**) A schematic diagram of MoS_2 structure. The red and black lines in the left panel indicate unit cell c_1 and supercell c_2 of MoS_2, respectively. The right panel presents their corresponding first Brillouin zones (BZ). **K** point (in red) in BZ of c_1 are folded to **K'** point (in black) in BZ of c_2. If the supercell repeats along b axis, we can obtain supercell c_3 marked by the blue rectangle, and its corresponding BZ would be folded to near **ΓX** line along the armchair direction. Furthermore, armchair nanoribbon (ANR) can be obtained by introducing vacuum space along b axis, which suppresses the period potential of supercell c_3 along b axis. Consequently, BZ of nanoribbon degenerates into **ΓX** line, and **K** and **Q** points in BZ of c_1 are folded into **Γ** and **X** point, respectively. Electronic band structure of 1L MoS_2 (**b**) and 1L 39ANR (**c**), where the Fermi level is set at zero and the fat bands with the projected weight of Mo atomic d_{z^2} and $d_{x^2-y^2}$ orbitals within the eigenstates. Charge density isosurfaces of electron states of 1L MoS_2 (**d**) and 1L39ANR (**e**), specified in **b** and **c**, respectively.

3.3 Electronic band structures and Fermi levels of nanoribbons with different widths

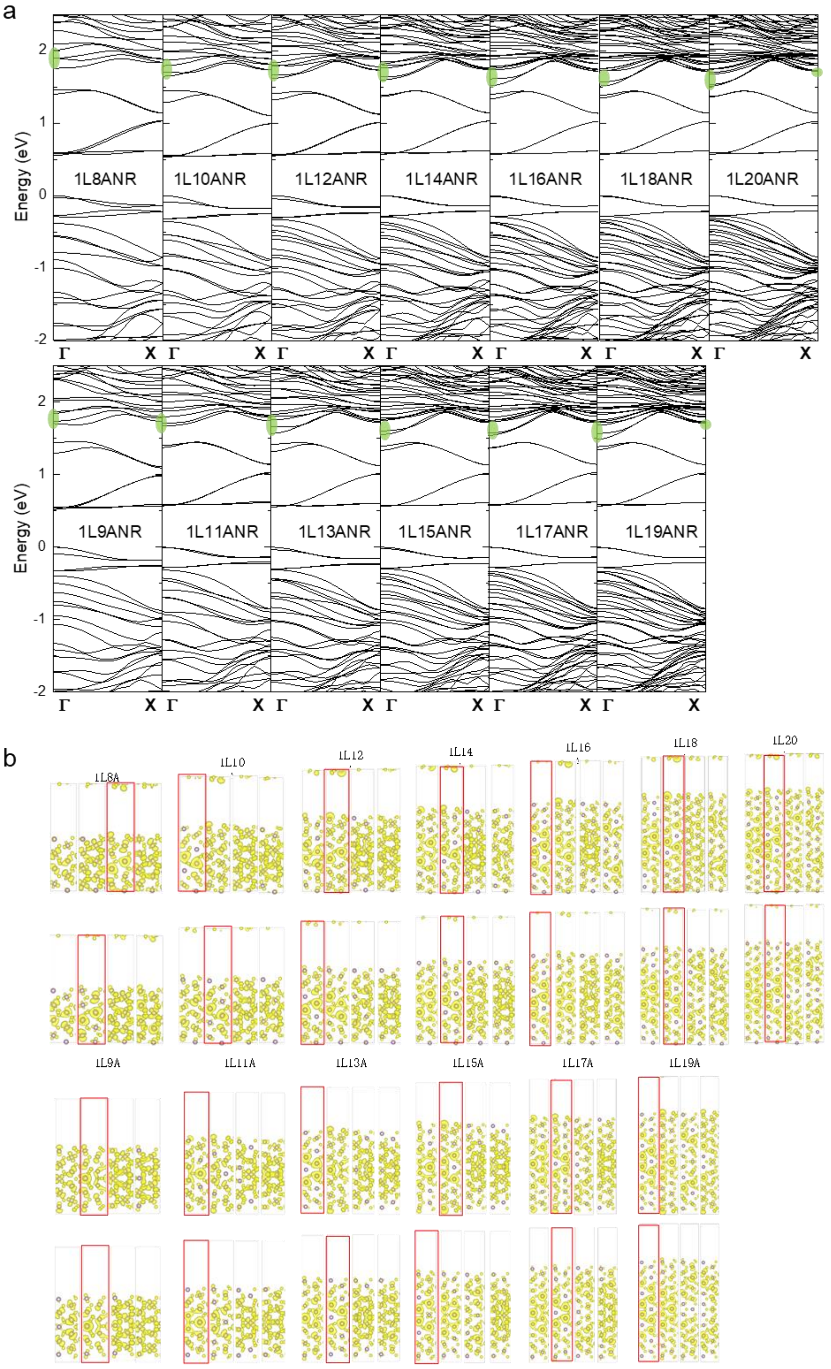

Supplementary Figure 9 Band structures (**a**) of 1L ANR with width n=8-20, and their corresponding charge density isosurfaces of electron states above edge bands at **K** and **Q** points (**b**), indicated by green region. The four charge density isosurfaces for a certain 1L ANR at **K** or **Q** point are arranged in energy ascending sort order. And the red rectangles indicate the charge density of determined $c_{\mathbf{K}}$ and $c_{\mathbf{Q}}$ states.

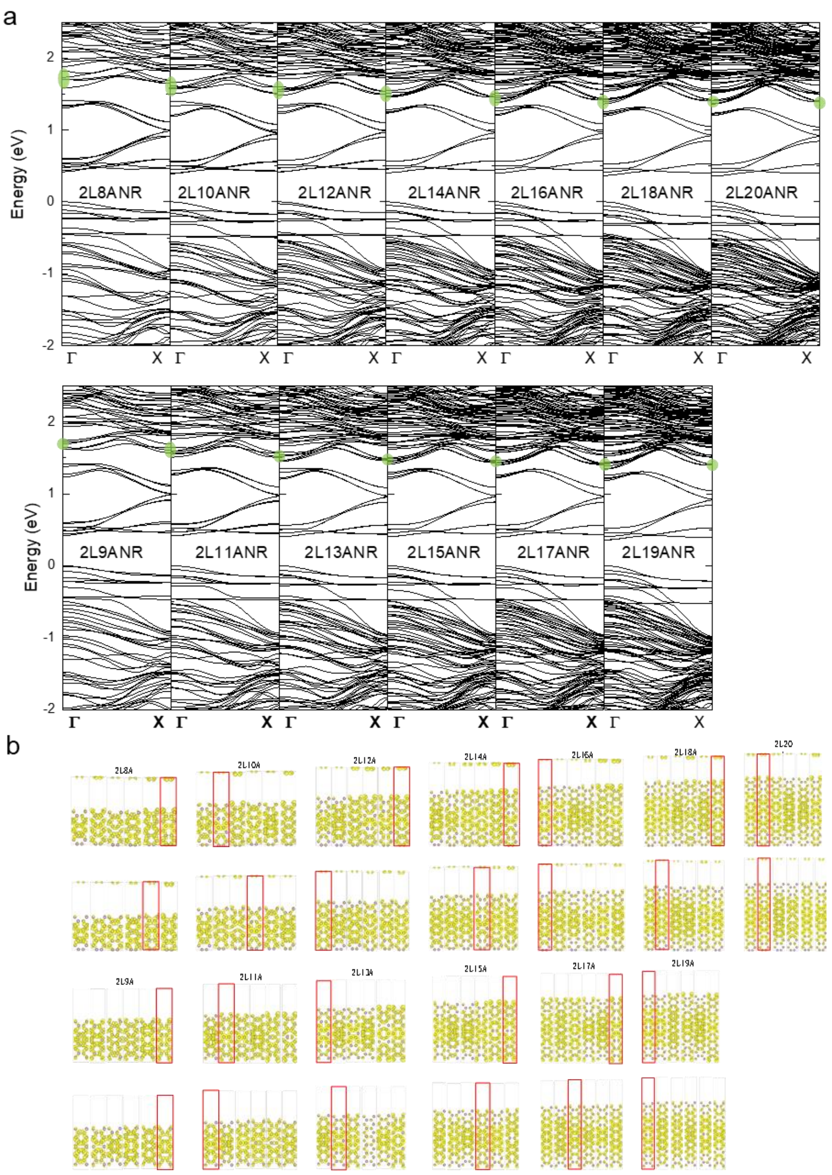

Supplementary Figure 10 Band structures (**a**) of 2L ANR with width n=8-20, and their corresponding charge density isosurfaces of electron states above edge bands at **K** and **Q** points (**b**), indicated by green region. The six charge density isosurfaces for a certain 2L ANR at **K** or **Q** point are arranged in energy ascending sort order. And the red rectangles indicate the charge density of determined $c_{\mathbf{K}}$ and $c_{\mathbf{Q}}$ states.

To investigate the edge effect on the electronic states involved in the DRR process, we further calculated the band structures of 1L and 2L ANR with varying width (10-30 Å, n=8-20), as shown in Supplementary Fig. 9 and Supplementary Fig. 10, respectively. The obtained band gap of 1L ANR as a function of nanoribbon width, presented in Supplementary Fig. 11a, agrees with that in the previous report[9]. To further identify folded $c_{\mathbf{K}}$ and $c_{\mathbf{Q}}$ states in ANR, we plot charge density of electron states at **Γ** and **X** points, specified in the corresponding band structure. Compared with the

charge density, the folded $c_{\mathbf{K}}$ and $c_{\mathbf{Q}}$ states involved in LA(**M**)+TA(**M**) mode of 1L and 2L ANR are identified and their energy as a function of nanoribbon width are plotted in Supplementary Fig. 11b and c, respectively. The difference between $c_{\mathbf{K}}$ and $c_{\mathbf{Q}}$ states are also presented in Supplementary Fig. 11d, which has a close relationship with the intensity of LA(**M**)+TA(**M**) mode. At the edge, the band bending is expected, which have been directly observed by scanning tunneling microscopy and spectroscopy (STM/S)[10]. The edge band profiles represent the electrostatic potential and can be calculated by solving the equations of semiconductor statistics together with Poisson's equation for the relationship between potential and charge[7, 11]. Assuming a space charge layer with a constant density ρ extending from the edge to a point at a distance *d* from the edge and being zero beyond *d*. Poisson's equation can be written as $\frac{\mathrm{d}^2\phi}{\mathrm{d}z^2} = -\frac{4\pi\rho}{4\pi\varepsilon_0\varepsilon_0}$, where ϕ and ε_0 are the electrostatic potential and the static dielectric constant, respectively. By solving the Poisson's equation:

$$\phi(\mathrm{z}) = \begin{cases} \frac{2\pi\rho}{4\pi\varepsilon_0\varepsilon_0}(z-\mathrm{d})^2, & \mathrm{z} \le \mathrm{d} \\ 0 & , \mathrm{z} > \mathrm{d} \end{cases} \quad (5)$$

Where the *d* indicates the length of space charge layer and *z* is the distance from the edge. The obtained $\phi(0)$ represents the total band bending. The band bending results from edge induced energy variation of electron states. Here, we assume that energy bending for different states have the same *d*, but different total bending degrees, which agrees with STS results that VBM and CBM exhibit obvious different total bending[10]. Consequently, $E_{\mathbf{CK}}$-$E_{\mathbf{CQ}}$ as a function of nanoribbon widths (w) can be fitted by $E_{\mathbf{CK}}$-$E_{\mathbf{CQ}}$ = $2A(\mathrm{w-2d})^2$, as presented by dash lines in the Supplementary Fig. 11d, where *A* results from different bending for $c_{\mathbf{K}}$ and $c_{\mathbf{Q}}$ states. Considering ANR with two edges, we approximate that edge effect on $E_{\mathbf{CK}}$-$E_{\mathbf{CQ}}$ in nanoribbon is twice as large as that at the single edge of MoS_2, and equivalent distance from the edge is half of the nanoribbon width. Therefore, the edge effect on $E_{\mathbf{CK}}$-$E_{\mathbf{CQ}}$ can be obtained as $E_{\mathbf{CK}}$-$E_{\mathbf{CQ}}$ = $A(\mathrm{z-d})^2$, plotted in the Supplementary Fig. 12. $E_{\mathbf{CK}}$-$E_{\mathbf{CQ}}$ at 2L basal plane is much larger than that at 1L basal plane indicates large possibility of LA(**M**)+TA(**M**) mode in 2L basal plane. When approaching the edge, $E_{\mathbf{CK}}$-$E_{\mathbf{CQ}}$ increases, indicating enhancement of LA(**M**)+TA(**M**) mode at the edge. From the Supplementary Fig. 12, the possibility of DRRS process of LA(**M**)+TA(**M**) mode at 1L MoS_2 is much lower than that at 2L MoS_2, leading to the absence of LA(**M**)+TA(**M**) mode at the basal plane and edge of 1L MoS_2. Furthermore, from the Supplementary Fig. 12, we can obtain the length for $E_{\mathbf{CK}}$-$E_{\mathbf{CQ}}$ varying near

edge induced by band bending is about 1.8 nm. Since the very weak DRRS signal (396 cm^{-1}) can already be observed on the basal plane of 2L MoS_2 (Fig. 3b red spectrum), the increase of E_{CK}-E_{CQ} enhances LA(**M**)+TA(**M**) mode. Therefore, the intensity variation region of LA(**M**)+TA(**M**) mode induced by edge effect is estimated to be 1.8 nm.

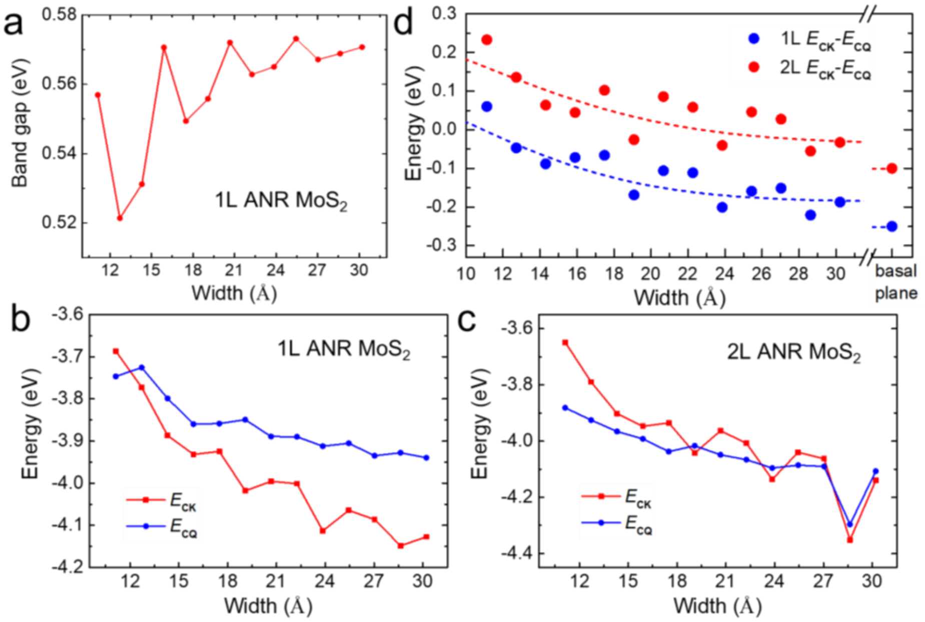

Supplementary Figure 11 Calculated electronic band energy as a function of the nanoribbon width. (**a**) The band gap of 1L ANR, the energy of folded c_K and c_Q states in 1L (**b**) and 2L (**c**) ANR, and (**d**) the energy difference between c_K and c_Q states in 1L and 2L ANR as a function of nanoribbon width n.

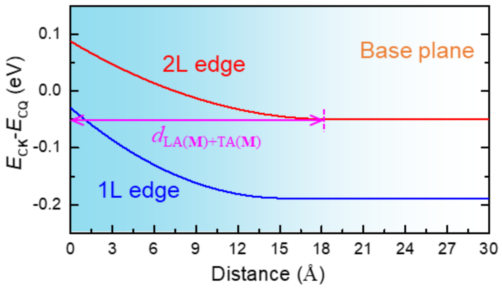

Supplementary Figure 12 The energy difference between c_K and c_Q states (E_{CK}-E_{CQ}) in 1L and 2L ANR as a function of the nanoribbon width.

We further calculated the work functions as a function of nanoribbon width to investigate the charge transfer for alignment between the edge and the basal plane. We first calculated the electrostatic potential of MoS_2 and ANR with a series of width, and 1L 20ANR is taken as an

example to illustrate the approach to obtain the vacuum level (E_{vac}). Supplementary Fig. 13a presents the electrostatic potential energy as a function of the distance from MoS_2 center along c axis, and the E_{vac} can be obtained as electrostatic potential energy away from MoS_2. The work function (E_W) can be obtained by $E_W=E_{vac}-E_F$, where E_F is the Fermi level obtained from the band structure calculation. E_W as a function of ribbon widths is presented in Supplementary Fig. 13b, where E_W of ANR is obviously larger than that of the basal plane, indicating the charge transfer from the edge to the basal plane when they are aligned. This charge transfer results in depletion of electrons near the edge, which leads to the increase of the intensity of A_{1g} mode near the edge.

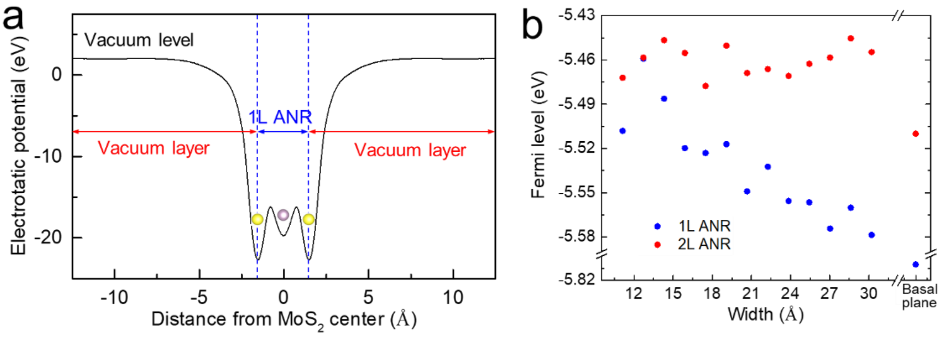

Supplementary Figure 13 Calculated Fermi level as a function of ribbon width. (**a**) The electrostatic potential energy of 1L 20ANR as a function of the distance from MoS_2 along the *c* axis. (**b**) The Fermi level referred to the vacuum level, i.e., the work function, of 1L and 2L ANR as a function the ribbon width.

3.4 Calculation of peak position of A_{1g} mode

To calculate the frequency of A_{1g} mode for different MoS_2 structures., we employed density functional perturbation theory (DFPT) using VASP code to calculate phonon frequency at **Γ** point of different types of MoS_2 and calculate Raman intensity ($\alpha_{xx}^2+\alpha_{xy}^2+\alpha_{yx}^2+\alpha_{yy}^2$) with Raman off-resonant activity calculator[12]. We then calculated the pristine MoS_2 (in supercells repeated along the armchair direction), ZNR (with width of 25.1 Å, 30.5 Å, and 35.9 Å), and ANR (with width of 15.6 Å, 17.4 Å, and 20.5 Å). The mode with a similar atomic displacement to the A_{1g} mode of the nanoribbon is denoted as A_{1g}-like mode. The mode with vibration localized at the edge is indicated in blue and the ones on the basal plane near the edge are indicated in orange (Supplementary Fig. 14).

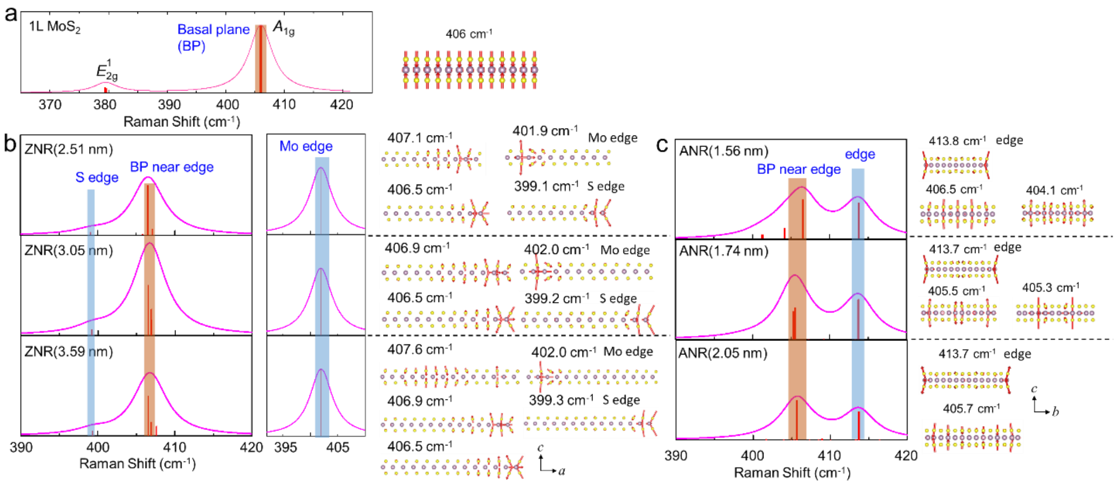

Supplementary Figure 14 Calculated Raman spectra and lattice vibration of 1L pristine MoS_2 (**a**), ZNR (**b**) and ANR (**c**) with different widths, where vibrational modes are obtained with VASP and Raman intensities are obtained with the Raman off-resonant activity calculator using VASP as a back end. Envelopes of each Raman spectra are evaluated by smearing the peaks with a half width of 5 cm^{-1}. Note that the Raman intensities near the Mo and S edge is no comparable.

For ZNR, there are S and Mo edge. Raman intensities near the two edge are not comparable, and their Raman spectra are presented separately in Supplementary Fig. 14b. According to calculated Raman intensities, there are three A_{1g}-like modes, with vibration localized at S edge (~399 cm^{-1}), Mo edge (~402 cm^{-1}), and with vibration component on basal plane near edge (~406.5 cm^{-1}). The frequencies of all these three A_{1g}-like modes exhibit negligible difference at different nanoribbon widths, indicating the decoupling between two edges. The higher frequency modes at ~407 cm^{-1} in ZNR show a low intensity, and the mode might result from band folding and splitting. It is obvious that the zigzag edge (no matter Mo or S edge) has a lower frequency than that of the basal plane, agreeing with the downshift of A_{1g} mode in experiment.

For ANR, there are two A_{1g}-like vibration modes localized related to the edge and basal plane. The former has higher frequency (~413 cm^{-1}) than the latter (~406 cm^{-1}), as presented in Supplementary Fig. 14c.

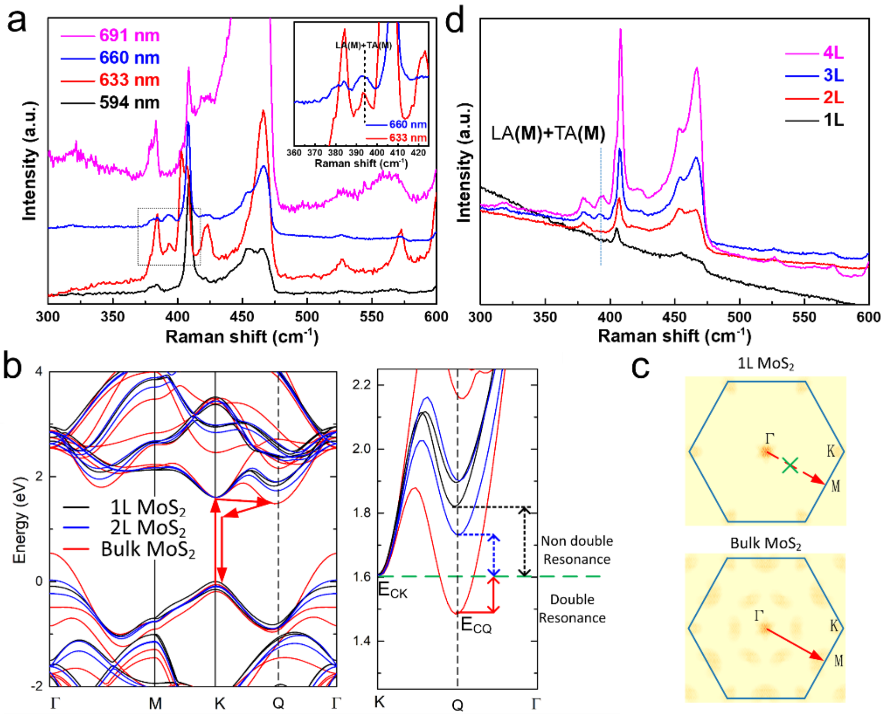

Supplementary Figure 15 Double-resonance Raman scattering (DRRS) of MoS_2. (a) Raman spectra of bulk MoS_2 excited by different laser lines. Laser power: 0.2 mW. **(b) Left panel**: the electronic band structure of 1L- (black), 2L- (blue) and bulk (red) MoS_2. As the layer number increases, the conduction band state at **Q** lowers until it aligns with the conduction band minimum at the **K** point. The red arrows in the left panel indicate the DRRS process. When the excitation energy is chosen to match or be near to an optical transition bandgap of MoS_2, the Raman intensity can be enhanced by the so-called resonant Raman effect. As shown in Supplementary Fig. 15b, the DRRS process starts with an incoming photon (660 nm laser), whose energy matches the bandgap near **K** point, creating an electron-hole pair near **K** point. The electron can be excited by a resonant transition from the state in the valence band to the state in the conduction band. Then the excited electron can be resonantly scattered by emitting a phonon with wave vector $\mathbf{q}_\mathbf{M}$ to the electron state in the conduction band near **Q** point when the energy of the **K** conduction band (E_{CK}) is larger than that of the **Q** conduction band (E_{CQ}). Afterward, the electron is inelastically scattered back to the virtual electron state near the **K** valley by emitting a second phonon with wave vector $-\mathbf{q}_\mathbf{M}$, where the electron-hole pair recombines and emits a photon with energy E_{laser} - $\hbar\omega_{LA(\mathbf{M})+TA(\mathbf{M})}$ in valence band near **K** points. Therefore, the Raman intensity of this phonon can be greatly enhanced[13]. However, if the E_{CQ} is larger than E_{CK}, the excited electron near the **K** valley will be scattered to a virtual state and then recombines with the hole in valence band near **K** points, resulting in the low

probability of the activation of phonon with wave vector $\mathbf{q}_M$. **Right panel:** Zoom-in curves of the state at the **Q** point of the left panel. (**c**) Density of states of phonons that satisfy the DRRS conditions for 1L and bulk MoS_2. The DRRS intensity is in arbitrary units. (**d**) Resonant Raman spectra of MoS_2 with different layers obtained with 660 nm laser line. Laser power: 0.2 mW. Acquisition time: 10 s. For the bulk or multilayer MoS_2, E_{CK} is much higher than E_{CQ} (Supplementary Fig. 15b), allowing the activation of the DRRS process (Supplementary Fig. 15c, lower panel) and appearance of the 396 cm^{-1} peak in the multilayer and bulk MoS_2 (upper two spectra). With the decrease of the layer number to bilayer or monolayer, E_{CK} becomes smaller than E_{CQ} (Supplementary Fig. 15b) and the DRRS process can no longer be activated (Supplementary Fig. 15c, upper panel). Therefore, the 396 cm^{-1} peak cannot be observed in the monolayer and bilayer MoS_2 (lower two spectra).

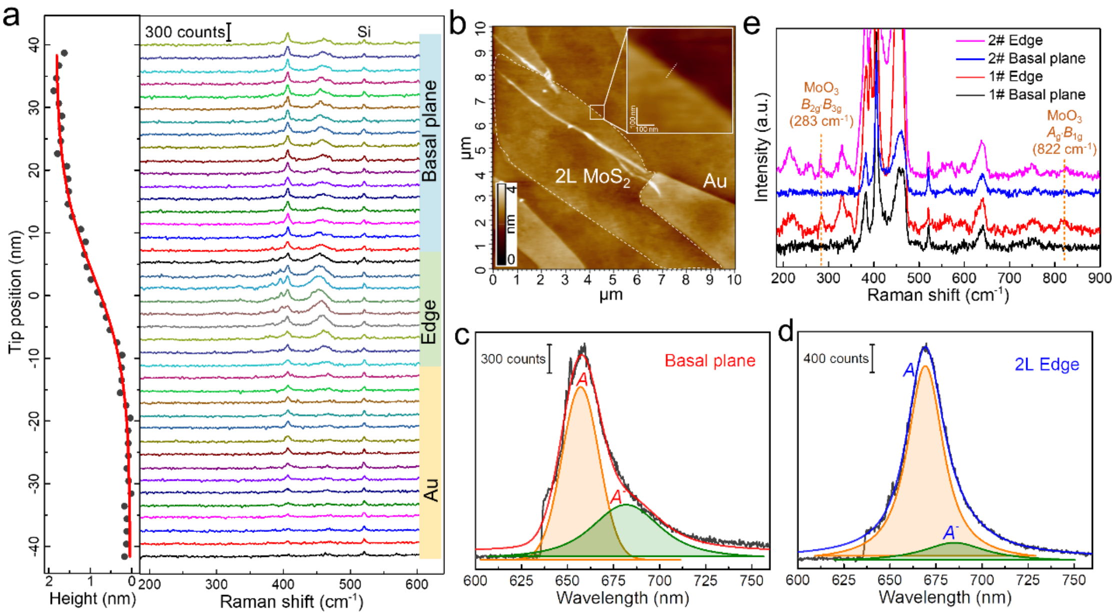

Supplementary Figure 16 Nanoscale characterization of bilayer edge in MoS_2. (**a**) Left panel: topographic height profile of the edge of bilayer MoS_2 marked in **b** inset. Right panel: the corresponding line-trace TERS spectra of the edge. Scan rate: 2 nm per step. The tip was scanned at a velocity of 2 nm s^{-1} and TERS spectra were acquired simultaneously with a laser power of 0.2 mW and an acquisition time of 0.4 s. Note that these spectra are original TERS signals without any data processing. (**b**) AFM image of the bilayer MoS_2 on an Au substrate. Inset: the zoom-in AFM image at the bilayer edge. (**c, d**) spectra of the bilayer MoS_2 in the basal plane and edge, respectively. This experiment was performed on an upright STM-TERS setup using an Ag STM tip. The

excitation laser wavelength is 632.8 nm. (**e**) Raman spectra of MoO_3 at the bilayer edge. The observation of MoO_3 in the edge site indicates that the edge is more chemically active than the basal plane and can be easily chemisorbed by the oxygen species. Note that these are the pure near field spectra after subtracting the far field signals.

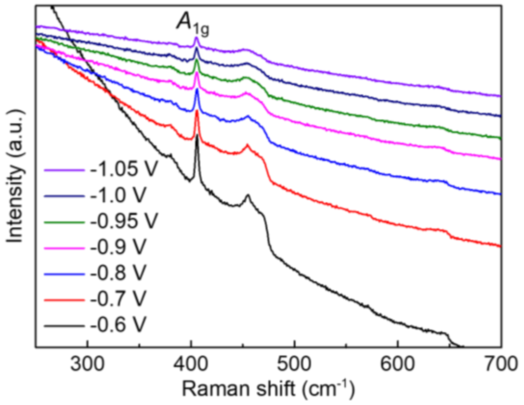

Supplementary Figure 17 EC-Raman spectra of the monolayer MoS_2. The three-electrode system was employed, namely, MoS_2-Au working electrode, the Pt reference electrode, and the Pt wire counter electrode. 0.5 M H_2SO_4 solution was used as the electrolyte. With the positive moving of the potential, all the Raman peaks increase due to the decrease of the charge screening effect. Noteworthy, the variation of A_{1g} mode intensity is remarkable, demonstrating the sensitivity of A_{1g} intensity to the electron density.

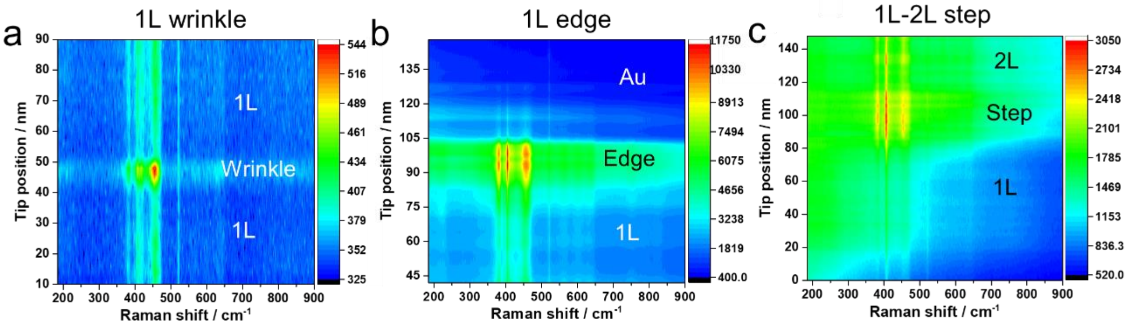

Supplementary Figure 18 Color-coded intensity map of the line-trace TERS image of three types of 1D defects. (**a**) 1L wrinkle. (**b**) 1L edge. (**c**) 1L-2L step. All the TEPL and Raman signals in the defect are stronger than that on the basal plane, indicating the defects have lower electron densities.

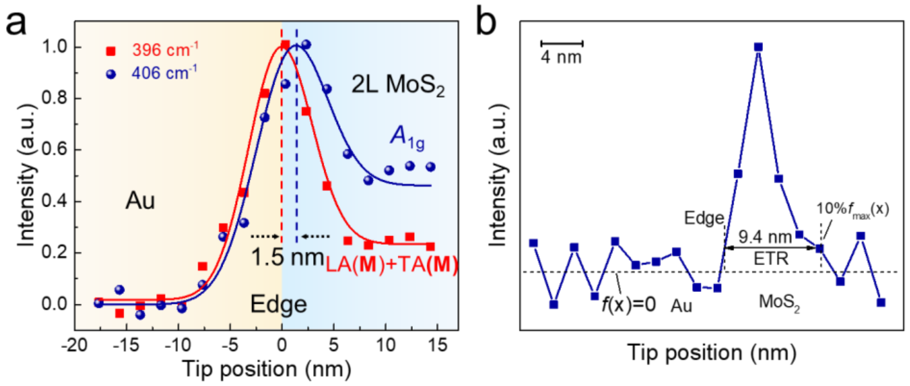

Supplementary Figure 19 TERS intensity profiles of another bilayer edge. (**a**) Plots of normalized intensities of two TERS peaks (396 and 406 cm^{-1}) of bilayer MoS_2 with the tip position. The solid lines are the fitted results. Note that these are the pure near field signals after subtracting the far field signals. The corresponding line-trace TERS spectra are shown in Supplementary Fig. 20a. (**a**) Enhanced TERS intensity profiles of the electronic transition region induced by bilayer edge after the deconvolution of the EM field intensity distribution.

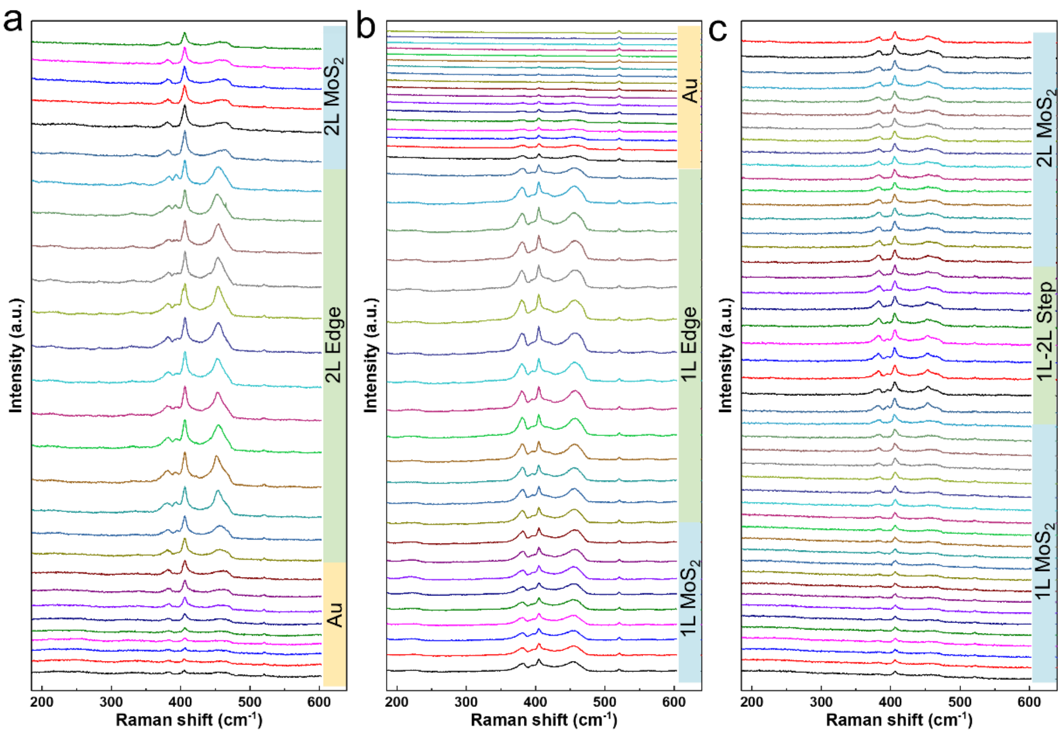

Supplementary Figure 20 Line-trace TERS spectra across the bilayer edges (a), monolayer edge (b), and step between monolayer and bilayer (c). Scan rate: 2 nm per step. The tip was scanned at a velocity of 2 nm s^{-1} and TERS spectra were acquired simultaneously. Note that these spectra are original TERS signals without any data processing.

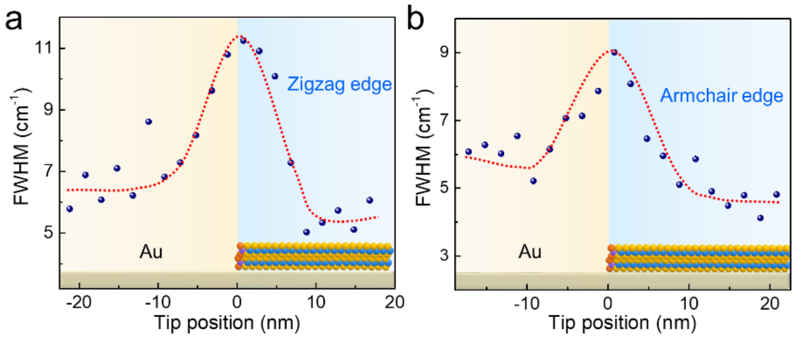

Supplementary Figure 21 Effect of the edge structure on the linewidth of the Raman A_{1g} mode. Plots of FWHM with the tip position for the zigzag edge (**a**) and the armchair edge (**b**). The dash red lines are guides for the eye.

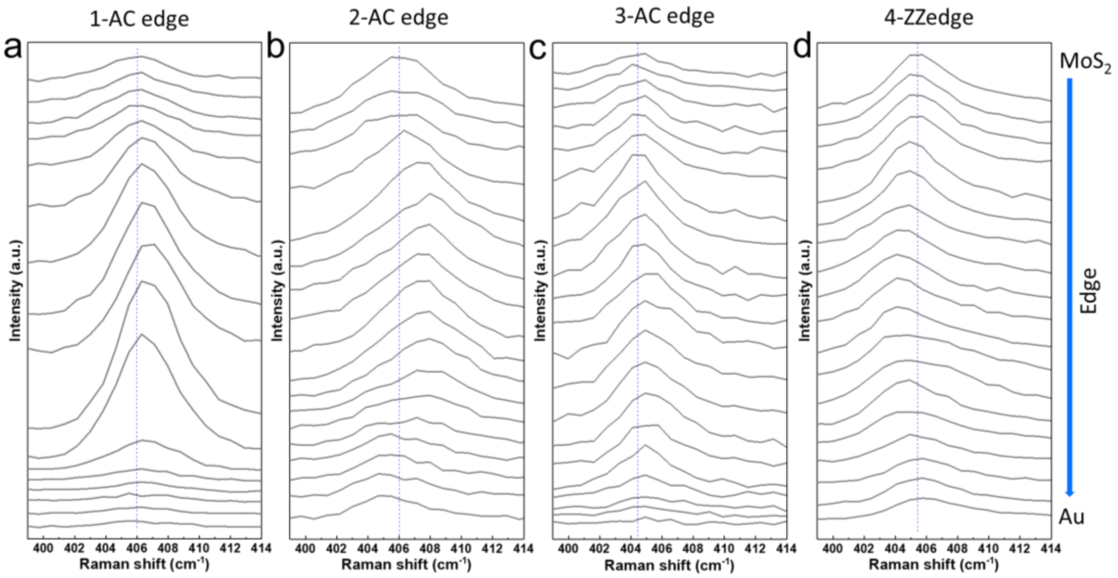

Supplementary Figure 22 Line-trace TERS spectra across four monolayer edges marked in Fig. 5d. Scan rate: 2 nm per step. The tip was scanned at a rate of 2 nm s^{-1} and TERS spectra were acquired simultaneously. Note that these spectra are original TERS signals without any data processing.

References

1. Su, W., Kumar, N., Mignuzzi, S., Crain, J. & Roy, D. Nanoscale mapping of excitonic processes in single-layer MoS_2 using tip-enhanced photoluminescence microscopy. *Nanoscale* **8**, 10564-10569 (2016).
2. Zhang, R. et al. Chemical mapping of a single molecule by plasmon-enhanced Raman scattering. *Nature* **498**, 82-86 (2013).
3. Zhong, J.H. et al. Probing the electronic and catalytic properties of a bimetallic surface with 3 nm resolution. *Nat. Nanotechnol.* **12**, 132-136 (2017).
4. Wang, X. et al. Tip-enhanced Raman spectroscopy for surfaces and interfaces. *Chem. Soc. Rev.* **46**, 4020-4041 (2017).

5. Zhu, C.R. et al. Strain tuning of optical emission energy and polarization in monolayer and bilayer MoS_2. *Phys. Rev. B* **88**, 121301 (2013).
6. Wang, Y., Cong, C., Qiu, C. & Yu, T. Raman spectroscopy study of lattice vibration and crystallographic orientation of monolayer MoS_2 under uniaxial strain. *Small* **9**, 2857-2861 (2013).
7. Yu, P.Y. & Cardona, M. Fundamentals of semiconductors: physics and materials properties (Springer, 2010).
8. Lee, B.G. et al. Quasi-Direct Optical Transitions in Silicon Nanocrystals with Intensity Exceeding the Bulk. *Nano Lett.* **16**, 1583-1589 (2016).
9. Li, Y., Zhou, Z., Zhang, S. & Chen, Z. MoS_2 Nanoribbons: High Stability and Unusual Electronic and Magnetic Properties. *J. Am. Chem. Soc.* **130**, 16739-16744 (2008).
10. Zhang, C., Johnson, A., Hsu, C.-L., Li, L.-J. & Shih, C.-K. Direct imaging of band profile in single layer MoS_2 on graphite: quasiparticle energy gap, metallic edge states, and edge band bending. *Nano Lett.* **14**, 2443-2447 (2014).
11. Zhang, C. et al. Visualizing band offsets and edge states in bilayer–monolayer transition metal dichalcogenides lateral heterojunction. *Nat. Commun.* **7**, 10349 (2016).
12. Bae, S. et al. Defect-Induced Vibration Modes of Ar^+-Irradiated MoS_2. *Phys. Rev. Appl.* **7**, 024001 (2017).
13. Carvalho, B.R. et al. Intervalley scattering by acoustic phonons in two-dimensional MoS_2 revealed by double-resonance Raman spectroscopy. *Nat. Commun.* **8**, 14670 (2017).
